# Add-on effect of kinesiotape in patients with acute lateral ankle sprain: a randomized controlled trial

**DOI:** 10.1186/s13063-020-4111-z

**Published:** 2020-02-12

**Authors:** Jeong-Cheol Shin, Jae-Hong Kim, Dongwoo Nam, Gwang-Cheon Park, Jeong-Soon Lee

**Affiliations:** 1 Department of Acupuncture and Moxibustion Medicine, College of Korean Medicine, DongShin University, Naju City, 58245 Republic of Korea; 2 Clinical Research Center, DongShin University Gwangju Korean Medicine Hospital, 141, Wolsan-ro, Nam-gu, Gwangju City, 61619 Republic of Korea; 3 Department of Acupuncture and Moxibustion Medicine, College of Korean Medicine, KyungHee University, Seoul, 02447 Republic of Korea; 4 Department of Nursing, Christian College of Nursing, Gwangju City, 61662 Republic of Korea

**Keywords:** Acupuncture, Ankle sprain, Kinesiotape, Randomized controlled trial

## Abstract

**Background:**

Evidence for the add-on effect of kinesiotape (KT) with acupuncture for treating ankle sprains remains insufficient. We assessed the add-on effect of KT on ankle sprains by comparing acupuncture combined with KT (AcuKT) with acupuncture alone in patients with acute lateral ankle sprain (ALAS).

**Methods:**

This study was a multicenter, randomized controlled clinical trial that included a per-protocol analysis of the add-on effect of KT on ALAS. The randomization was software based and only the assessors were blinded. Sixty participants (20 each from three centers) with grade I or II ALAS were randomly assigned to acupuncture (*n* = 30) or AcuKT (*n* = 30) groups. Both groups received acupuncture treatment once daily, 5 days per week for 1 week. The AcuKT group received additional KT treatment. Visual analog scale (VAS) scores for pain and the Foot and Ankle Outcome Score (FAOS) were obtained, and edema measurements were performed at baseline (week 0), at the end of the intervention (week 1), and at 4 weeks after intervention (week 5). The European Quality of Life Five Dimension-Five Level Scale (EQ-5D-5 L) measurements were conducted at week 0, week 1, week 5, and week 26 after the intervention. The number of recurrent ankle sprains was determined at 4, 8, 12 and 26 weeks after the intervention.

**Results:**

Fifty-six patients with ALAS completed the trial (AcuKT group, *n* = 27; acupuncture group, *n* = 29). There were significant changes in visual analog scale score (AcuKT, *P* < 0.001; acupuncture, *P* < 0.001), the FAOS (AcuKT, *P* < 0.001; acupuncture, *P* < 0.001), and EQ-5D-5 L measurements (AcuKT, *P* < 0.001; acupuncture, *P* < 0.001) within both groups. There were no significant differences between groups in terms of any outcome or in a subanalysis based on symptom severity.

**Conclusions:**

These results indicate that AcuKT did not show a positive add-on effect of KT with acupuncture in terms of pain reduction, edema, recovery of function, activities of daily living, quality of life or relapse of ALAS.

**Trial registration:**

Clinical Research Information Service (cris.nih.go.kr), KCT0002257. Registered on 27 February 2017.

## Background

Acute ankle sprain is an acute injury to one or more ankle ligaments [1]. The most frequent ankle injury is ligament sprain, with up to 85% cases involving the lateral ligament complex [2]. Ankle sprains are commonly considered to be benign injuries that resolve quickly [3]; however, if not managed appropriately, patients may experience recurrent instability, chronic pain, osteochondral lesions of the talus, premature osteoarthritis and other significant long-term disabilities [4]. Ankle sprain has a high incidence, with a consequent high prevalence of persistent problems that lead to high costs to society because of increased use of health care resources and an inability to work [5].

The three major types of treatment for ankle sprain are surgery, immobilization with a plaster cast or splint, and functional treatment with bandage, tape, different brace, or balance training [6]. Management of acute ankle sprains typically involves rest, ice compression, elevation and functional rehabilitation. In more severe cases, the ankle is normally immobilized for a few days, and the patient must use crutches to avoid weight bearing on the injured ankle [7].

In 2017, ankle sprain was the fifth most common reason for visits to Korean medicine clinics, and 1 million Korean patients with ankle sprain received Korean medicine treatment [8]. In addition to conventional treatments for ankle sprain, complementary and alternative therapies such as acupuncture, herbs, cupping therapy, taping and *chuna* were used to relieve pain, reduce swelling and help the body restore damaged tissue [9].

Kinesiotape (KT), developed in the 1970s by the Japanese chiropractor K. Kase, is an elastic therapeutic tape used for the treatment of sports injuries and a variety of other conditions [10]. KT differs from the traditional white athletic tape because of the wave-like grain design on its adhesive surface [11]. First, the specialized grain and elasticity of KT provides a tensile force to the skin which is purported to lift the fascia and soft tissue, allowing mobility while providing therapeutic benefits [12]. Second, KT is air-permeable and water resistant and can be worn for several days without removal [13]. The application of KT has been suggested to result in an improvement in muscle contractility by supporting weakened muscles, decreasing inflammation and pain by increasing lymph and blood flow, and increasing the range of motion of the joint by adjusting the misalignment of muscle fibers, myofascia and joints [14, 15]. KT may also assist in the management of ankle sprain by reducing pain, altering muscle function, improving circulation, enhancing proprioception and repositioning subluxed joints [16].

Although KT is mainly used with acupuncture for the treatment of ankle sprain in Korean medicine, evidence regarding the add-on effect of KT with acupuncture for treating ankle sprains is insufficient. Therefore, this study was performed to investigate the add-on effect of KT with acupuncture in acute lateral ankle sprain (ALAS) by comparing acupuncture combined with KT (AcuKT) with acupuncture alone in patients with ALAS.

## Methods

This study followed the Standard Protocol Items: Recommendations for Interventional Trials (SPIRIT) and Consolidated Standards of Reporting Trials (CONSORT) statement (Additional file 1). The detailed methods of this study have been reported previously [17].

### Study design

This study was a prospective, outcome assessor-blinded, multicenter, randomized controlled trial with a 1:1 allocation ratio. Participants (*n* = 20 from each of the three centers) who met the inclusion criteria were randomly allocated to either the acupuncture group (*n* = 10 from each of the three centers) or AcuKT group (*n* = 10 from each of the three centers). Both groups received acupuncture treatment once daily, 5 days per week (excluding Saturday and Sunday) for 1 week, and the AcuKT group also received the ankle meridian tendino-musculature and eight-shape form of KT treatment. Outcome measures were determined at baseline (week 0), 1 week after the first intervention (week 1), and 4 weeks after completion of the intervention (week 5). The number of recurrent ankle sprains was determined at 4, 8, 12 and 26 weeks after the completion of the intervention. The study design is summarized in Table 1.

**Table 1:** Standard Protocol Items: Recommendations for Interventional Trials (SPIRIT) figure showing the enrollment, interventions, and data collection protocols

	STUDY PERIOD										
	Enrolment	Allocation	Post-allocation								Close-out
TIMEPOINT	Screening		Visit1	Visit2	Visit3	Visit4	Visit5	Visit6	Visit7	Visit8	Visit9
	Week		1					5	8	13	27
ENROLMENT											
Informed consent	X										
Sociodemographic profile	X										
Medical history	X										
Vital signs	X	X	X	X	X	X	X	X			
Inclusion/exclusioncriteria	X										
Allocation		X									
INTERVENTIONS											
Acupuncture treatment			X	X	X	X	X				
Acupuncture and Kinesio taping combination treatment			X	X	X	X	X				
ASSESSMENTS											
Change of medical history			X	X	X	X	X	X	X	X	X
Safety assessment			X	X	X	X	X	X			
Visual Analogue Scale of pain			X				X	X			
Foot and Ankle Outcome Score			X				X	X			
Edema of ankle sprain			X				X	X			
European Quality of Life Five Dimension-Five Level Scale			X				X	X			X
Number of recurrent ankle injuries								X	X	X	X

### Ethical considerations

This study was conducted in accordance with the Declaration of Helsinki, and the protocol of this study (version 1.0) was approved by the Institutional Review Board (IRB) of DongShin University Gwangju Korean Medicine Hospital (DSGOH-039; approval date 20 March 2017), DongShin University Mokpo Oriental Hospital (DSMOH-002; approval date 27 March 2017), and KyungHee Korean Medicine Hospital (KOMCIRB-161014-HR-057; approval date 28 April 2017) before the trial began. This trial was registered at the Clinical Research Information Service (cris.nih.go.kr; KCT0002257). The purpose and potential risks of this study were fully explained to the participants. All participants provided written informed consent before participating in this study.

### Participant recruitment

Participants were recruited at three hospitals in the Republic of Korea: DongShin University Gwangju Korean Medicine Hospital, DongShin University Mokpo Oriental Hospital and KyungHee Korean Medicine Hospital. This study was publicized via local newspapers, the internet, and posters in communities and hospitals. The clinical research coordinator continuously monitored the medical conditions of enrolled participants to maximize adherence to the intervention protocols.

### Participation

Potential participants aged >19 years who had sustained a grade I or II ALAS within the past 7 days and who voluntarily signed the informed consent form were included in the study. Grade I ankle sprain was defined as no loss of function, no ligamentous laxity (i.e., negative anterior drawer and talar tilt tests), little or no hemorrhaging, no point tenderness, total ankle motion reduced by ≤5° and swelling ≤0.5 cm. Some loss of function, a positive anterior drawer test (anterior talofibular ligament involvement), a negative talar tilt test (no calcaneofibular ligament involvement), hemorrhaging, point tenderness, decreased total ankle motion >5° but <10°, and swelling >0.5 cm but <2.0 cm were characteristics of grade II ankle sprain [18].

Potential participants whose general condition was unsatisfactory or who were unfit for acupuncture or AcuKT therapies were excluded. The detailed exclusion criteria were: 1) fracture as confirmed by radiography, or a grade III ankle sprain; near total loss of function, positive anterior drawer and talar tilt tests, hemorrhaging, extreme point tenderness, total ankle motion reduction >10°, or swelling >2.0 cm (considered as grade III ankle sprain) [18]; 2) history of fracture in the same ankle during the previous year; 3) a wound or skin disease at the KT attachment site; 4) serious disease (e.g., cancer, kidney disease, liver disease, disease of the central nervous system, dementia, blood clotting disturbance such as hemophilia and so forth); 5) motor or sensory disturbance caused by a nervous system disorder in the leg with the sprain; and 6) pregnancy or breastfeeding.

### Randomization and blinding

After obtaining baseline measurements, SPSS version 20.0 (SPSS Inc., Chicago, IL, USA) was used to assign a serial number to the 60 participants and to randomly allocate 30 of them to each group. The serial number codes were inserted into opaque envelopes that were sealed and kept in a double-locked cabinet and opened in the presence of the participant and a guardian.

We could only adopt a single outcome assessor-blinding approach because sham treatment was impossible due to the characteristics of KT application, which included attachment to the skin. During the study, the assessor was blinded to group assignments, and data analysts without conflicts of interest were involved in this study.

### Implementation

A clinical research coordinator generated the allocation sequence, enrolled the participants, and assigned participants to the interventions.

### Intervention

For the acupuncture treatment, sterile, stainless steel, disposable acupuncture needles (size 0.25 × 30 mm; Dong Bang Acupuncture, Inc., Boryeong, Republic of Korea; product no. A84010.02) with guide tubes were vertically inserted into the ST36, ST41, BL60, BL62, KI3, KI6, GB39 and GB40 acupuncture points on the affected side [9]. The depth of insertion was 10–20 mm, depending on the location of the needle [19]. After insertion, the needles were left in position for 15 min for every session. Manual stimulation and electroacupuncture were not applied.

**Fig. 1:**
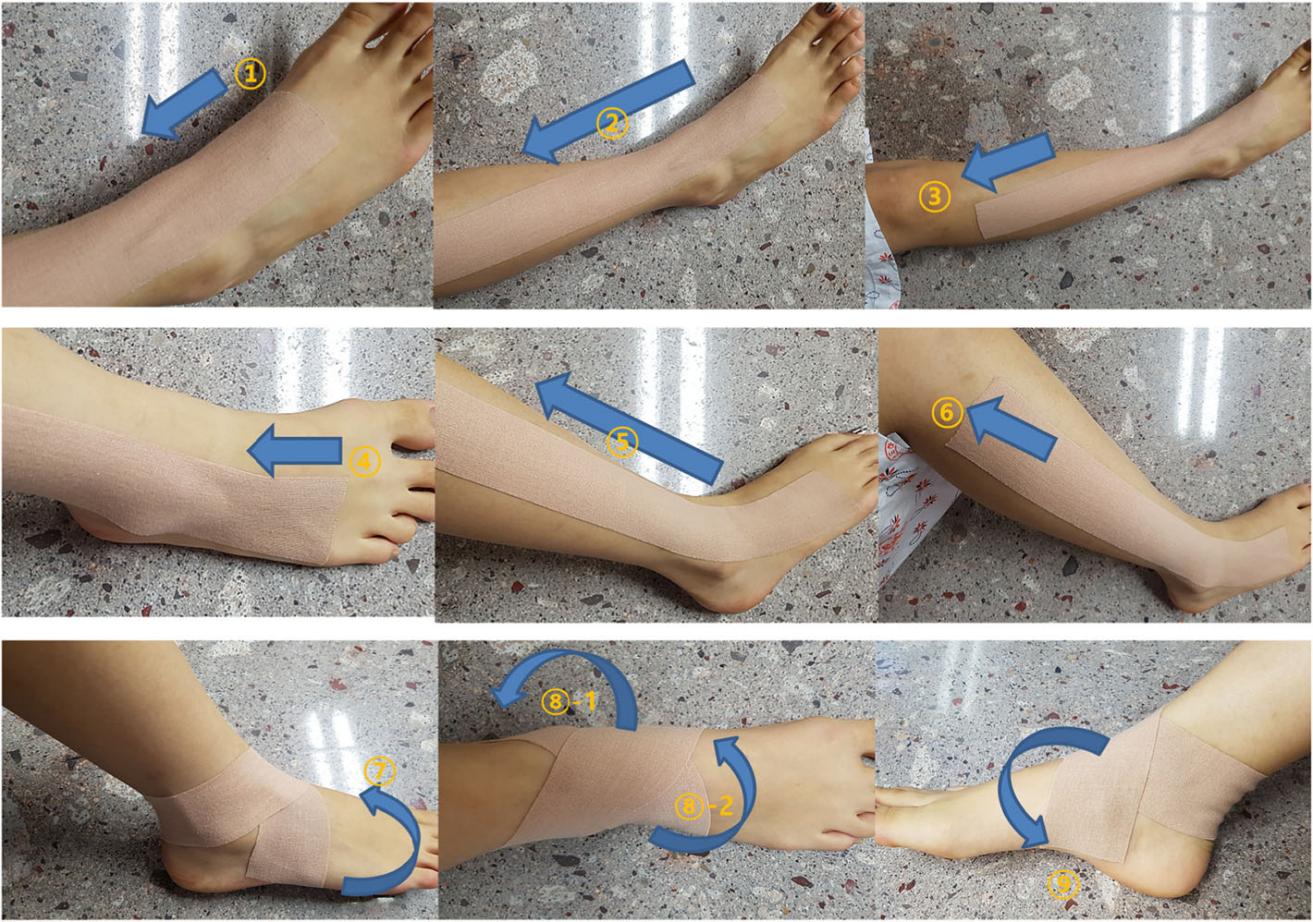
Application of kinesiotape. Steps 1–3: An I-shaped tape is applied from ST42 to ST36 over the tibialis anterior muscle. Steps 4–6: An I-shaped tape is applied from GB42 to GB34 over the peroneus longus and brevis muscles. Steps 7–9: An I-shaped tape is applied from the abductor digiti minimi muscle and wrapped around the ankle in a figure-of-eight shape to the abductor halluces muscle, covering both the medial and lateral malleoli

KT treatment was conducted after acupuncture treatment by the same practitioner. First, an I-shaped tape was applied from ST42 to ST36 over the tibialis anterior muscle (Fig. 1, steps 1–3). Second, an I-shaped tape was applied from GB42 to GB34 over the peroneus longus and brevis muscles (Fig. 1, steps 4–6). Third, an I-shaped tape was applied from the abductor digiti minimi muscle and was wrapped around the ankle in a figure-of-eight shape to the abductor halluces muscle, covering both the medial and lateral malleoli (Fig. 1, steps 7–9) [20]. NK-50 kinesiology tape was used (width 50 mm, thickness 0.5 mm; Nitto Denko Medical MFG Co., Ltd., Miyagi, Japan; product no. B07090.02). The tape was laid on the skin without being stretched, to prevent skin problems. The KT treatment was applied daily after removal of the tape applied the previous day, even in cases where the patient did not complain of itchiness [21].

### Outcome measurements

The primary outcome was the visual analog scale (VAS) score for pain, and the secondary outcomes were the Foot and Ankle Outcome Score (FAOS), edema, European Quality of Life Five Dimension-Five Level Scale (EQ-5D-5 L) scores, and the number of recurrent ankle sprains. VAS, FAOS, and edema measurements were performed at baseline (week 0; before intervention), 5 days after the first intervention (week 1; at the end of the intervention), and 4 weeks after the completion of the intervention (week 5). EQ-5D-5 L measurements were conducted at baseline, 5 days after the first intervention, 4 weeks after the completion of the intervention, and 26 weeks after the completion of the intervention (week 27). The numbers of recurrent ankle sprains were assessed at 4 (week 5), 8 (week 9), 12 (week 13) and 26 weeks after the completion of the intervention.

The primary outcome was the change in pain severity measured using a VAS pain scale. The VAS is a 10-cm straight line marked at each end with the anchor labels “no pain” and “pain as bad as it could be” [22]. Participants were asked to mark on the line a point representing the severity of their pain. Scores were recorded in millimeters, with a total score range of 0–100 mm [23].

The FAOS is a region-specific instrument that is intended to evaluate symptoms and functional limitation in individuals with generalized foot and ankle disorders. It is composed of the following five subscales: pain (9 items), other symptoms (7 items), activities of daily living (17 items), sports and recreational activities (5 items) and foot- and ankle-related quality of life (4 items). The subscales are scored separately using a Likert response format, with higher scores indicating higher levels of function [24].

Edema was measured in centimeters via the figure-of-eight method. The measuring tape was applied across the following landmarks in a figure-of-eight fashion: 1) navicular tuberosity; 2) distal tip of the lateral malleolus; 3) distal tip of the medial malleolus; and 4) base of the fifth metatarsal. The resulting value was compared with the corresponding value for the healthy ankle [25].

The EQ-5D is a generic instrument for assessing health-related quality of life. It is based on a descriptive system that defines health in terms of five dimensions: mobility, self-care, usual activities, pain/discomfort and anxiety/depression. Each dimension has three response categories corresponding to no problems, some problems, and extreme problems. The EQ-5D-5 L is a new version of EQ-5D that includes five levels of severity in each of the existing five EQ-5D dimensions [26].

Ankle sprain recurrence was defined as an ankle sprain occurring as a result of sports participation or other daily activities, and which caused one or more of the following: 1) stoppage of the sports activity; 2) limited participation in the next planned sports activity; 3) inability to go to work/school the next day; or 4) the need for medical attention (ranging from onsite care administered by a general practitioner to personal care administered by a sports physician) [27].

### Sample size calculation

In accordance with a previous study [28], we established the number of groups as two and the effect size as 0.906, with a one-sided alpha level of 0.025 and a statistical power of 0.8. Based on these parameters, the required sample size was 42 (21 per group). Estimating a maximum dropout rate of 30%, we determined that a total of 60 participants were required. The sample size calculation was detailed in our study protocol [17].

### Statistical analyses

With the approval of the IRB, the statistical analysis was revised from the study protocol. We performed per-protocol analyses for the assessment of efficacy and a supplementary full analysis set. Missing values were implemented by the last observation carried forward method. We compared the results of per-protocol analyses and analyses of the full analysis set. If there was a significant difference between the per-protocol and full analysis groups, the cause was reviewed and reflected during efficacy assessment. Analysis was performed by blinded biostatisticians with SPSS version 20.0 software (SPSS Inc., Chicago, IL, USA) using two-sided significance tests with a 5% significance level. Continuous variables are presented as means and standard deviations, and categorical variables are presented as count frequencies and percentages.

Baseline data were collected and compared using the independent *t* test, chi-squared test, and Fisher’s exact test. Differences between all outcome value changes in the two groups were compared via Wilcoxon signed-rank test and repeated-measures analysis of variance (ANOVA) (Friedman tests). Values of VAS, edema, EQ-5D-5 L, and FAOS were compared by repeated-measures ANOVA across two to three testing time points (week 0, week 1 and week 5). Differences between two groups of outcome value changes (week 0 versus week 1 and week 0 versus week 5) were compared with the Mann–Whitney *U* test (nonparametric test). Differences between the two groups in terms of number of recurrent ankle sprains (week 5, week 9, week 13 and week 27) were compared with the Mann–Whitney *U* test (nonparametric test). In accordance with the severity of ankle sprain, participants were divided into grade I and grade II groups. A subanalysis was conducted to investigate the differences in changes in VAS, edema, EQ-5D-5 L and FAOS (week 0 versus week 1 and week 0 versus week 5) between the two groups in the grade I and grade II group.

## Results

### Participants

**Fig. 2:**
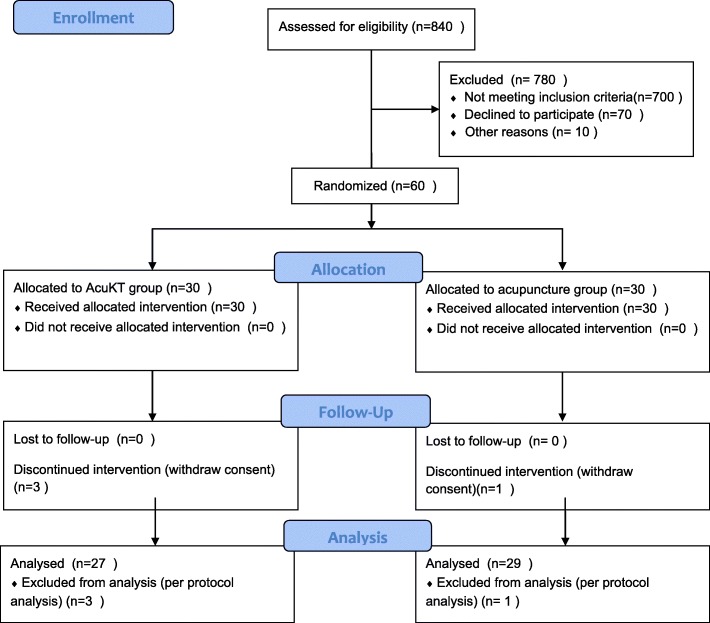
Consolidated Standards of Reporting Trials (CONSORT) 2010 flow diagram. AcuKT acupuncture combined with kinesiotape

We recruited participants between 28 April 2017 and 15 October 2018. During the study period, 840 patients were assessed for eligibility and 780 were excluded. Sixty patients were included in this study and were randomly assigned to an acupuncture group (*n* = 30) or an AcuKT group (*n* = 30). Three patients did not complete the treatment in the AcuKT group, while one did not complete treatment in the acupuncture group. The results of the per-protocol analysis for the assessment of efficacy were not different from those of the full analysis set. Thus, data for 56 participants with ankle sprains (AcuKT group, *n* = 27; acupuncture group, *n* = 29) were used in the final analysis (Fig. 2).

### Baseline characteristics

The baseline demographic characteristics and study variables of the 56 participants in the two groups are presented in Table 2. No significant differences in the baseline demographic characteristics or study variables were detected between the two groups (*P* > 0.05; Table 2).

**Table 2:** Homogeneity tests for baseline demographic characteristics and study variables of 56 participants with acute lateral ankle sprain

Dependent variables	AcuKT (*n* = 27)	Acupuncture (*n* = 29)	*t* test or chi-squared test (*P*)
	Mean ± SD or *n* (%)	Mean ± SD or *n* (%)	
Age (years)	39.81 ± 15.02	39.28 ± 14.23	0.14 (0.891)^a^
Gender (female)	17 (63.0%)	20 (69.0%)	0.23 (0.635)^b^
Lesion side (right)	14 (51.9%)	19 (65.5%)	1.09 (0.299)^b^
Sprain injury rating (grade I)	14 (46.7)	16 (55.2)	0.27 (0.599)^b^
Duration	3.30 ± 1.92	3.72 ± 1.62	0.90 (0.370)^b^
Body mass index (kg/m^2^)	24.78 ± 3.74	24.36 ± 3.55)	0.44 (0.663)^a^
VAS score of pain	4.11 ± 1.40	4.79 ± 2.02	1.44 (0.154)^a^
Degree of edema (cm)	0.56 ± 0.64	0.48 ± 0.78	−0.38 (0.706^)a^
EQ-5D-5 L	9.41 ± 2.37	9.90 ± 2.83	0.70 (0.488)^a^
FAOS	322.11 ± 65.78	318.32 ± 76.72	−0.21 (0.838^)a^
FAOS symptom/rigidity	68.01 ± 14.46	69.91 ± 16.52	0.46 (0.650)^a^
FAOS ache	69.39 ± 15.52	68.24 ± 17.18	−0.26 (0.794)^a^
FAOS function everyday life	75.93 ± 14.75	77.38 ± 16.17	0.35 (0.729)^a^
FAOS features sports/leisure	50.56 ± 25.34	52.17 ± 19.99	0.23 (0.818)^a^
FAOS quality of life	58.22 ± 21.23	50.63 ± 25.35	−1.19 (0.233)^a^

^a^*t* test; ^b^chi-squared test

*AcuKT* acupuncture combined with kinesiotape, *EQ-5D-5 L* European Quality of Life Five Dimension-Five Level Scale, *FAOS* Foot and Ankle Outcome Score, *SD* standard deviation, *VAS* visual analog scale

### Efficacy of primary and secondary outcomes

After 1 week of intervention we observed significant improvements in the AcuKT group and acupuncture group for changes in VAS pain, EQ-5D-5 L, total FAOS, FAOS symptom/rigidity, FAOS ache, FAOS function everyday life, FAOS features sports/leisure, and FAOS quality of life scores (Table 3).

**Table 3:** Changes in outcome measures (week 0 versus week 1, week 0 versus week 5) after treatment completion between patients who received AcuKT (*n* = 27) and those who received acupuncture (*n* = 29) for acute lateral ankle sprain

Groups	Dependent variables	Week 0 (mean ± SD)	Week 1 (mean ± SD)	Week 5 (mean ± SD)	Difference (week 1 – week 0)	Z (*P*)^a^	Difference (week 5 – week 0)	Z (*P*)^a^	Chi-squared test (*P*)^b^
AcuKT group (*n* = 27)	Degree of edema	0.56 ± 0.64	0.26 ± 1.59	0.19 ± 0.43	−0.30 ± 0.54	−2.53 (0.011)	−0.36 ± 0.68	−2.43 (0.015)	4.87 (0.088)
	VAS score of pain	4.11 ± 1.40	1.89 ± 1.44	0.97 ± 1.04	−2.21 ± 1.58	−4.42 (<0.001)	−3.14 ± 1.34	4.55 (<0.001)	44.83 (<0.001)
	Total EQ-5D-5 L	9.41 ± 2.37	7.19 ± 1.94	6.04 ± 1.45	−2.22 ± 3.06	−3.29 (0.001)	− 3.37 ± 2.68	4.02 (<0.001)	30.42 (<0.001)
	Total FAOS	322.11 ± 65.78	402.44 ± 65.785	439.65 ± 50.27	80.33 ± 62.74	−4.54 (<0.001)	117.54 ± 61.29	4.54 (<0.001)	45.66 (<0.001)
	FAOS symptom/rigidity	68.01 ± 14.46	81.44 ± 12.16	89.01 ± 10.97	13.43 ± 12.82	−3.92 (<0.001)	21.00 ± 16.70	4.11 (<0.001)	28.95 (<0.001)
	FAOS ache	69.39 ± 15.52	84.41 ± 11.75	91.92 ± 8.96	15.02 ± 13.59	−4.07 (<0.001)	22.53 ± 14.57	4.46 (<0.001)	41.16 (<0.001)
	FAOS function everyday life	75.93 ± 14.75	90.03 ± 10.32	95.00 ± 6.71	14.09 ± 13.20	−4.14 (<0.001)	19.07 ± 13.90	4.36 (<0.001)	33.88 (<0.001)
	FAOS features sports/leisure	50.56 ± 25.34	74.27 ± 17.24	84.43 ± 14.57	23.72 ± 20.09	−4.05 (<0.001)	33.87 ± 20.47	4.55 (<0.001)	41.57 (<0.001)
	FAOS quality of life	58.22 ± 21.23	72.29 ± 23.30	79.28 ± 16.16	14.07 ± 22.22	−2.96 (0.003)	21.06 ± 21.43	3.94 (<0.001)	18.37 (<0.001)
Acupuncture group (*n* = 29)	Degree of edema	0.48 ± 0.78	0.24 ± 0.74	0.26 ± 0.57	−0.24 ± 0.64	−1.94 (0.052)	−0.22 ± 0.60	−1.31 (0.191)	5.48 (0.065)
	VAS score of pain	4.79 ± 2.02	2.31 ± 1.86	1.58 ± 1.89	−2.47 ± 1.86	−4.53 (<0.001)	−3.21 ± 1.99	4.46 (<0.001)	39.41 (<0.001)
	Total EQ-5D-5 L	9.90 ± 2.83	7.76 ± 2.32	7.00 ± 2.19	−2.14 ± 2.50	−4.00 (<0.001)	−2.90 ± 2.54	−4.22 (<0.001)	34.24 (<0.001)
	Total FAOS	318.32 ± 76.72	385.00 ± 77.90	416.25 ± 74.24	66.67 ± 49.51	−4.33 (<0.001)	97.92 ± 73.06	−4.27 (<0.001)	31.74 (<0.001)
	FAOS symptom/rigidity	69.91 ± 16.52	82.10 ± 13.94	86.30 ± 13.51	12.19 ± 12.38	−3.79 (<0.001)	16.39 ± 14.59	−4.13 (<0.001)	21.56 (<0.001)
	FAOS ache	68.24 ± 17.18	80.90 ± 16.14	86.17 ± 16.33	12.66 ± 11.09	−4.20 (<0.001)	17.93 ± 15.55	−4.15 (<0.001)	26.26 (<0.001)
	FAOS function everyday life	77.38 ± 16.17	87.06 ± 13.84	92.37 ± 10.98	9.68 11.05	−3.77 (<0.001)	14.99 ± 14.05	−4.43 (<0.001)	28.71 (<0.001)
	FAOS features sports/leisure	52.17 ± 19.99	70.52 ± 20.63	79.66 ± 17.83	18.35 18.75	−3.88 (<0.001)	27.49 ± 22.91	−4.23 (<0.001)	30.14 (<0.001)
	FAOS quality of life	50.63 ± 25.35	64.41 ± 24.78	71.76 ± 24.30	13.79 ± 21.42	−3.11 (<0.001)	21.13 ± 22.93	−3.80 (<0.001)	20.33 (<0.001)

^a^Wilcoxon signed-rank test; ^b^Repeated-measures analysis of variance (Friedman test)

*AcuKT* acupuncture combined with kinesiotape, *EQ-5D-5 L* European Quality of Life Five Dimension-Five Level Scale, *FAOS* Foot and Ankle Outcome Score, *SD* standard deviation, *VAS* visual analog scale

Repeated-measures ANOVA showed no significant interaction between time and group with respect to all study variables (Table 4).

**Table 4:** Results of repeated-measures analysis of variance for the outcomes of treatment between patients who received AcuKT (*n* = 27) and those who received acupuncture (*n* = 29) for acute lateral ankle sprain

Dependent variables	Group (*n*)	Week 0 (mean ± SD)	Week 1 (mean ± SD)	Week 5 (mean ± SD)	Source	SS	df	Mean square	F	*p*
VAS score of pain	Acu (*n* = 29)	4.79 ± 2.02	1.62 ± 0.62	1.58 ± 1.89	Time	303.30	1.66	182.28	125.67	<0.001
	AcuKT (*n* = 27)	4.11 ± 1.40	1.56 ± 0.64	0.97 ± 1.04	Group×time	0.50	1.66	0.30	0.21	0.774
Degree of edema	Acu (*n* = 29)	0.48 ± 0.78	2.31 ± 1.86	0.26 ± 0.57	Time	2.97	2	1.48	8.98	<0.001
	AcuKT (*n* = 27)	0.56 ± 0.64	1.90 ± 1.45	0.19 ± 0.43	Group×time	0.14	2	0.07	0.41	0.662
Total EQ-5D-5 L	Acu (*n* = 29)	9.90 ± 2.83	7.76 ± 2.32	7.00 ± 2.19	Time	288.60	1.61	179.50	49.33	<0.001
	AcuKT (*n* = 27)	9.41 ± 2.37	7.19 ± 1.94	6.04 ± 1.45	Group×time	1.79	1.61	1.11	0.305	0.689
Total FAOS	Acu (*n* = 29)	318.32 ± 76.72	385.00 ± 77.90	416.25 ± 74.24	Time	338,926.08	1.56	169,463.04	107.76	<0.001
	AcuKT (*n* = 27)	322.11 ± 65.78	402.44 ± 60.10	439.65 ± 50.27	Group×time	2827.45	1.56	18,100.11	0.90	0.389
FAOS symptom/rigidity	Acu (*n* = 29)	69.91 ± 16.52	82.10 ± 13.94	86.30 ± 13.51	Time	10,221.41	1.69	6064.62	58.88	<0.001
	AcuKT (*n* = 27)	68.01 ± 14.46	81.44 ± 12.16	89.01 ± 10.97	Group×time	159.70	1.69	94.76	0.92	0.388
FAOS ache	Acu (*n* = 29)	68.24 ± 17.18	80.90 ± 16.14	86.17 ± 16.33	Time	11,962.24	1.53	7832.55	78.00	<0.001
	AcuKT (*n* = 27)	69.39 ± 15.52	84.41 ± 11.75	91.92 ± 8.96	Group×time	147.87	1.53	96.82	0.96	0.365
FAOS function everyday life	Acu (*n* = 29)	77.38 ± 16.17	87.06 ± 13.84	92.37 ± 10.98	Time	8533.93	1.57	5428.96	61.82	<0.001
	AcuKT (*n* = 27)	75.93 ± 14.75	90.03 ± 10.32	95.00 ± 6.71	Group×time	168.90	1.57	107.45	1.22	0.292
FAOS features sports/ leisure	Acu (*n* = 29)	52.17 ± 19.99	70.52 ± 20.63	79.66 ± 17.83	Time	27,533.87	1.55	17,764.44	81.35	<0.001
	AcuKT (*n* = 27)	50.56 ± 25.34	74.27 ± 17.24	84.43 ± 14.57	Group×time	329.04	1.55	212.29	0.97	0.363
FAOS quality of life	Acu (*n* = 29)	50.63 ± 25.35	64.41 ± 24.78	71.76 ± 24.30	Time	12,868.12	2	6364.06	28.09	<0.001
	AcuKT (*n* = 27)	58.22 ± 21.23	72.29 ± 23.30	79.28 ± 16.16	Group×time	0.96	2	1.97	0.002	0.998

*Acu* acupuncture, *AcuKT* acupuncture combined with kinesiotape, *df* degrees of freedom, *EQ-5D-5 L* European Quality of Life Five Dimension-Five Level Scale, *FAOS* Foot and Ankle Outcome Score, *SD* standard deviation, *SS* sum of squares, *VAS* visual analog scale

There were no significant differences in all variables between the two groups with respect to VAS pain, edema, EQ-5D-5 L, and FAOS (week 0 versus week 1, week 0 versus week 5, and week 1 versus week 5) (Table 5).

**Table 5:** Comparison of changes in outcome measurements between patients who received AcuKT (*n* = 27) and those who received acupuncture (*n* = 29) for acute lateral ankle sprain

Dependent variables	Group (*n*)	Week 0 (mean ± SD)	Difference (week 1 – week 0)	Z (*P*)^a^	Difference (week 5 – week 0)	Z (*P*)^a^	Difference (week 5 – week 1)	Z (*P*)^a^
VAS score of pain	Acu (*n* = 29)	4.79 ± 2.02	−2.47 ± 1.86	−0.25 (0.804)	−3.21 ± 1.99	−0.52 (0.603)	− 0.74 ± 1.23	−0.72 (0.471)
	AcuKT (*n* = 27)	4.11 ± 1.40	−2.21 ± 1.58		−3.14 ± 1.34		− 0.93 ± 1.07	
Degree of edema	Acu (*n* = 29)	0.48 ± 0.78	−0.24 ± 0.64	−0.50 (0.620)	− 0.22 ± 0.60	−1.11 (0.269)	0.02 ± 0.44	−0.87 (0.386)
	AcuKT (*n* = 27)	0.56 ± 0.64	−0.30 ± 0.54		− 0.36 ± 0.68		− 0.07 ± 0.52	
Total EQ-5D-5 L	Acu (*n* = 29)	9.90 ± 2.83	−2.14 ± 2.50	−0.11 (0.914)	− 2.90 ± 2.54	−0.95 (0.341)	− 0.76 ± 1.81	−0.58 (0.561)
	AcuKT (*n* = 27)	9.41 ± 2.37	−2.22 ± 3.06		−3.37 ± 2.68		−1.15 ± 1.66	
Total FAOS	Acu (*n* = 29)	318.32 ± 76.72	66.67 ± 49.51	−0.30 (0.762)	97.92 ± 73.06	−0.71 (0.476)	31.25 ± 40.88	−0.86 (0.389)
	AcuKT (*n* = 27)	322.11 ± 65.78	80.33 ± 62.74		117.54 ± 61.29		37.21 ± 41.42	
FAOS symptom/rigidity	Acu (*n* = 29)	69.91 ± 16.52	12.19 ± 12.38	−0.67 (0.948)	16.39 ± 14.59	−0.97 (0.332)	4.19 ± 10.65	−1.05 (0.295)
	AcuKT (*n* = 27)	68.01 ± 14.46	13.43 ± 12.82		21.00 ± 16.70		7.57 ± 11.04	
FAOS ache	Acu (*n* = 29)	68.24 ± 17.18	12.66 ± 11.09	−0.49 (0.622)	17.93 ± 15.55	−1.03 (0.301)	5.27 ± 9.16	−0.85 (0.396)
	AcuKT (*n* = 27)	69.39 ± 15.52	15.02 13.59		22.53 ± 14.57		7.51 ± 8.68	
FAOS function everyday life	Acu (*n* = 29)	77.38 ± 16.17	9.68 11.05	−0.85 (0.398)	14.99 ± 14.05	−1.31 (0.189)	5.31 ± 10.05	−0.33 (0.736)
	AcuKT (*n* = 27)	75.93 ± 14.75	14.09 13.20		19.07 ± 13.90		4.98 ± 6.32	
FAOS features sports/leisure	Acu (*n* = 29)	52.17 ± 19.99	18.35 18.75	−0.87 (0.382)	27.49 ± 22.91	−0.86 (0.388)	9.14 ± 12.11	−0.35 (0.728)
	AcuKT (*n* = 27)	50.56 ± 25.34	23.72 ± 20.09		33.87 ± 20.47		10.16 ± 13.59	
FAOS quality of life	Acu (*n* = 29)	50.63 ± 25.35	13.79 ± 21.42	−0.03 (0.974)	21.13 ± 22.93	−0.17 (0.869)	7.34 ± 16.47	−0.23 (0.817)
	AcuKT (*n* = 27)	58.22 ± 21.23	14.07 ± 22.22		21.06 ± 21.43		6.99 ± 23.42	

^a^Mann–Whitney *U* test

*Acu* acupuncture, *AcuKT* acupuncture combined with kinesiotape, *EQ-5D-5 L* European Quality of Life Five Dimension-Five Level Scale, *FAOS* Foot and Ankle Outcome Score, *SD* standard deviation, *VAS* visual analog scale

A subanalysis based on the severity of ankle sprain showed no significant differences in all variables between the two groups in the grade I and grade II groups (Tables 6 and 7).

**Table 6:** Comparison of changes in outcome measurement between patients who received AcuKT (*n* = 13) and those who received acupuncture (*n* = 16) for grade I acute lateral ankle sprain

Dependent variables	Group (*n*)	Week 0 (mean ± SD)	Week 1 (mean ± SD)	Week 5 (mean ± SD)	Difference (week 1 – week 0)	Z (*P*)^a^	Difference (week 5 – week 0)	Z (*P*)^a^	Difference (week 5 – week 1)	Z (*P*)^a^
VAS score of pain	Acu (*n* = 16)	4.33 ± 1.82	2.32 ± 1.91	1.79 ± 2.07	−2.01 ± 1.96	−0.84 (0.400)	−2.54 ± 2.13	− 0.80 (0.426)	− 0.53 ± 1.38	−0.73 (0.463)
	AcuKT (*n* = 13)	4.34 ± 1.56	1.97 ± 1.85	1.12 ± 1.13	−2.37 ± 1.84		−3.08 ± 1.45		−0.85 ± 1.13	
Degree of edema	Acu (*n* = 16)	0.44 ± 0.89	0.31 ± 0.79	0.28 ± 0.74	−0.13 ± 0.72	−0.38 (0.704)	− 0.16 ± 0.64	−0.53 (0.600)	− 0.03 ± 0.26	−0.24 (0.893)
	AcuKT (*n* = 13)	0.23 ± 0.60	0.08 ± 0.49	0.11 ± 0.29	−0.15 ± 0.38		−0.11 ± 0.51		0.03 ± 0.51	
Total EQ-5D-5 L	Acu (*n* = 16)	9.19 ± 1.60	7.69 ± 2.47	6.81 ± 2.11	−1.50 ± 2.34	−0.45 (0.655)	−2.38 ± 2.45	−0.33 (0.739)	− 0.88 ± 1.96	−0.48 (0.630)
	AcuKT (*n* = 13)	8.77 ± 2.39	6.85 ± 2.08	6.31 ± 1.75	−1.92 ± 3.12		−2.50 ± 2.62		−0.54 ± 1.13	
Total FAOS	Acu (*n* = 16)	331.69 ± 43.64	395.62 ± 74.82	411.52 ± 81.83	63.93 ± 59.95	−0.44 (0.661)	79.83 ± 71.06	−0.44 (0.661)	15.90 ± 22.40	−0.66 (0.511)
	AcuKT (*n* = 13)	349.06 ± 43.64	429.66 ± 48.30	440.15 ± 48.82	81.37 ± 61.03		95.26 ± 43.07		10.49 ± 37.59	
FAOS symptom/rigidity	Acu (*n* = 16)	73.61 ± 11.58	83.67 ± 11.66	85.23 ± 13.18	10.06 ± 11.46	−0.88 (0.378)	11.61 ± 12.27	−1.92 (0.055)	1.56 ± 8.56	−1.24 (0.216)
	AcuKT (*n* = 13)	69.19 ± 14.04	84.03 ± 12.53	90.90 ± 8.20	14.84 ± 9.09		21.35 ± 13.48		6.87 ± 9.52	
FAOS ache	Acu (*n* = 16)	70.78 ± 13.97	83.47 ± 14.85	85.91 ± 18.06	12.69 ± 12.23	−0.07 (0.947)	15.13 ± 15.23	−0.04 (0.965)	2.44 ± 7.17	−0.58 (0.562)
	AcuKT (*n* = 13)	75.80 ± 16.68	88.22 ± 11.42	90.79 ± 10.43	12.42 ± 10.61		15.79 ± 12.04		2.56 ± 7.74	
FAOS function everyday life	Acu (*n* = 16)	79.36 ± 9.95	90.31 ± 11.54	90.88 ± 12.74	10.9 ± 9.79	−0.22 (0.826)	11.52 ± 10.87	−0.42 (0.677)	0.57 ± 3.54	−0.42 (0.673)
	AcuKT (*n* = 13)	79.48 ± 17.97	92.83 ± 10.15	94.66 ± 7.64	13.35 ± 14.40		14.33 ± 16.08		1.83 ± 5.41	
FAOS features sports/ leisure	Acu (*n* = 16)	52.50 ± 13.29	72.19 ± 19.06	78.44 ± 20.31	19.69 ± 22.02	−0.86 (0.390)	25.94 ± 24.51	−0.18 (0.860)	6.25 ± 9.75	−1.03 (0.304)
	AcuKT (*n* = 13)	54.62 ± 17.61	80.77 ± 17.89	83.08 ± 16.01	26.92 ± 17.97		29.23 ± 14.41		2.31 ± 12.18	
FAOS quality of life	Acu (*n* = 16)	55.45 ± 25.10	65.99 ± 26.21	71.08 ± 28.86	10.54 ± 22.56	−0.22 (0.826)	15.63 ± 24.36	−0.13 (0.895)	5.09 ± 8.60	−1.09 (0.278)
	AcuKT (*n* = 13)	69.98 ± 14.42	83.81 ± 20.45	80.73 ± 13.41	13.83 ± 23.40		12.79 ± 19.00		−3.08 ± 27.89	

^a^Mann–Whitney *U* test

*Acu* acupuncture, *AcuKT* acupuncture combined with kinesiotape, *EQ-5D-5 L* European Quality of Life Five Dimension-Five Level Scale, *FAOS* Foot and Ankle Outcome Score, *SD* standard deviation, *VAS* visual analog scale

**Table 7:** Comparison of changes in outcome measurements between patients who received AcuKT (*n* = 14) and those who received acupuncture (*n* = 13) for grade II acute lateral ankle sprain

Dependent variables	Group (*n*)	Week 0 (mean ± SD)	Week 1 (mean ± SD)	Week 5 (mean ± SD)	Difference (week 1 – week 0)	Z (*P*)^a^	Difference (week 5 – week 0)	Z (*P*)^a^	Difference (week 5 – week 1)	Z (*P*)^a^
VAS score of pain	Acu (*n* = 13)	5.35 ± 2.17	2.31 ± 1.89	1.32 ± 1.70	− 3.04 ± 1.61	−1.37 (0.170)	−4.03 ± 1.51	−1.73 (0.083)	−0.99 ± 1.01	−0.22 (0.825)
	AcuKT (*n* = 14)	3.90 ± 1.26	1.83 ± 1.01	0.83 ± 0.96	−2.07 ± 1.34		−3.07 ± 1.32		−1.00 ± 1.05	
Degree of edema	Acu (*n* = 13)	0.54 ± 0.66	0.15 ± 0.69	0.23 ± 0.27	−0.38 ± 0.51	−0.36 (0.720)	− 0.31 ± 0.56	−1.18 (0.240)	0.08 ± 0.60	−1.06 (0.287)
	AcuKT (*n* = 14)	0.86 ± 0.53	0.43 ± 0.65	0.27 ± 0.52	0.43 ± 0.65		−0.59 ± 0.75		−0.16 ± 0.53	
Total EQ-5D-5 L	Acu (*n* = 13)	10.77 ± 3.75	7.85 ± 2.23	7.23 ± 2.35	−2.92 ± 2.56	−0.79 (0.430)	−3.54 ± 2.60	−0.88 (0.378)	− 0.62 ± 1.66	−1.30 (0.193)
	AcuKT (*n* = 14)	10.00 ± 2.29	7.50 ± 1.83	5.79 ± 1.12	−2.50 ± 3.08		−4.21 ± 2.42		−1.71 ± 1.90	
Total FAOS	Acu (*n* = 13)	301.87 ± 104.02	371.92 ± 82.61	422.07 ± 66.53	70.05 ± 34.70	−0.05 (0.961)	120.20 ± 71.90	−0.44 (0.662)	50.15 ± 50.70	−1.02 (0.308)
	AcuKT (*n* = 14)	297.09 ± 63.25	377.16 ± 60.33	439.18 ± 53.42	78.74 ± 67.66		140.75 ± 68.84		62.01 ± 27.40	
FAOS symptom/rigidity	Acu (*n* = 13)	2.62 ± 0.87	80.18 ± 16.62	87.62 ± 14.33	14.82 ± 13.42	−0.63 (0.527)	22.26 ± 15.51	−0.37 (0.715)	7.44 ± 12.34	−0.20 (0.845)
	AcuKT (*n* = 14)	2.29 ± 0.61	79.04 ± 11.74	87.26 ± 13.10	12.13 ± 15.77		20.35 ± 19.42		8.22 ± 12.61	
FAOS ache	Acu (*n* = 13)	65.35 ± 20.69	77.75 ± 17.68	86.50 ± 14.65	12.62 ± 9.99	−0.75 (0.451)	21.38 ± 15.85	−1.31 (0.189)	8.75 ± 10.38	−1.10 (0.273)
	AcuKT (*n* = 14)	66.91 ± 15.28	80.87 ± 11.31	92.98 ± 7.59	17.43 ± 15.89		29.54 ± 13.37		12.11 ± 6.94	
FAOS function everyday life	Acu (*n* = 13)	65.12 ± 20.62	83.06 ± 15.78	94.21 ± 8.48	8.12 ± 12.65	−0.80 (0.423)	19.26 ± 16.65	−1.07 (0.285)	11.15 ± 12.38	−0.17 (0.865)
	AcuKT (*n* = 14)	63.44 ± 12.07	87.42 ± 10.15	95.32 ± 5.99	14.78 ± 12.49		22.68 ± 10.44		7.90 ± 5.81	
FAOS features sports/leisure	Acu (*n* = 13)	51.75 ± 26.67	68.46 ± 23.04	81.15 ± 14.88	16.71 ± 14.47	−0.39 (0.697)	29.40 ± 21.59	−1.07 (0.284)	12.69 ± 14.09	−1.10 (0.271)
	AcuKT (*n* = 14)	46.79 ± 23.42	68.24 ± 14.74	85.69 ± 13.57	21.46 ± 21.55		38.90 ± 24.48		17.44 ± 10.66	
FAOS quality of life	Acu (*n* = 13)	44.69 ± 25.36	62.48 ± 23.80	72.59 ± 18.32	17.78 ± 20.08	−0.34 (0.733)	27.90 ± 19.88	−0.05 (0.961)	10.12 ± 22.91	−0.57 (0.558)
	AcuKT (*n* = 14)	47.30 ± 21.06	61.59 ± 21.06	77.93 ± 18.77	14.29 ± 23.81		29.29 ± 22.52		16.34 ± 13.54	

^a^Mann–Whitney *U* test

*Acu* acupuncture, *AcuKT* acupuncture combined with kinesiotape, *EQ-5D-5 L* European Quality of Life Five Dimension-Five Level Scale, *FAOS* Foot and Ankle Outcome Score, *SD* standard deviation, *VAS* visual analog scale

There were no significant differences between the two groups regarding the number of recurrent ankle sprains (at week 5, week 9, week 13, and week 27) and the changes in EQ-5D-5 L (week 0 versus week 27) (Tables 8 and 9).

**Table 8:** Comparisons of number of recurrences at different time points between patients who received AcuKT (*n* = 27) and those who received acupuncture (*n* = 29) for acute lateral ankle sprain

Dependent variables	AcuKT (*n* = 27) (mean ± SD)	Acu (*n* = 29) (mean ± SD)	Z (*P*)^a^
Total relapse	0.33 ± 1.62	1.03 ± 2.86	−1.11 (0.268)
Week 5 relapse	−0.04 ± 1.06	0.31 ± 1.07	−1.33 (0.185)
Week 9 relapse	0.00 ± 0.48	0.34 ± 0.86	−1.44 (0.149)
Week 13 relapse	0.22 ± 0.64	0.31 ± 0.76	−0.31 (0.758)
Week 27 relapse	0.15 ± 0.82	0.07 ± 0.75	−0.62 (0.535)

^a^Mann–Whitney *U* test

*Acu* acupuncture, *AcuKT* acupuncture combined with kinesiotape

**Table 9:** Comparison of changes in EQ-5D-5 L scores from baseline to 26 weeks after treatment between patients who received AcuKT (*n* = 27) and those who received acupuncture (*n* = 29) for acute lateral ankle sprain

Dependent variables	Group (*n*)	Week 0 (mean ± SD)	Week 27 (mean ± SD)	Difference (week 27 – week 0)	Z (*P*)^a^
Total EQ-5D-5 L	Acu (*n* = 29)	9.90 ± 2.83	6.07 ± 2.48	−3.83 ± 3.43	−0.58
	AcuKT (*n* = 27)	9.41 ± 2.37	5.52 ± 1.37	−3.89 ± 2.79	(0.954)

^a^Mann–Whitney *U* test

*Acu* acupuncture, *AcuKT* acupuncture combined with kinesiotape, *EQ-5D-5 L* European Quality of Life Five Dimension-Five Level Scale

### Safety evaluation

Adverse events that occurred in this study were recorded on a case report form after evaluating their relationship with the intervention. No adverse events that were related to the intervention occurred in this study.

## Discussion

To the best of our knowledge, this is the first randomized controlled study to investigate the add-on effects of KT with acupuncture in terms of pain reduction, edema, recovery of function, activities of daily living, quality of life, and relapse of ALAS by comparing the effects of AcuKT with the effects of acupuncture alone. The design of our study (i.e., a 1-week duration of treatment, acupoint selected for acupuncture, and KT treatment method) was determined with reference to a previous study [1, 9, 28]. We observed significant improvements in the AcuKT group and acupuncture group (i.e., changes in VAS pain, EQ-5D-5 L, total FAOS, FAOS symptom/rigidity, FAOS ache, FAOS function everyday life, FAOS features sports/leisure, and FAOS quality of life scores).

There were two major findings in our study. First, AcuKT did not show any positive add-on effects of KT with acupuncture in terms of pain reduction, edema, recovery of function, activities of daily living, quality of life, or relapse of ALAS. Second, a subanalysis considering the severity of ankle sprain demonstrated no positive add-on effects of KT with acupuncture in terms of pain reduction, edema, recovery of function, activities of daily living, quality of life, or relapse of patients with ALAS.

There are several explanations for our results. First, there is conflicting evidence regarding the efficacy of KT in the prevention and management of ankle sprain. Three systematic reviews reported that KT demonstrated little clinical significance or effect on ankle movement and various measures of strength compared to usual care or sham tape [29–31]. However, a systematic review showed that the use of KT produced an immediate reduction in pain [29]. Furthermore, other systematic reviews reported that the use of KT produced small improvements in movement and muscle activity [32], and that KT could be used for the prevention and management of lateral ankle injuries [33]. Conflicting findings from these systematic reviews may be caused by insufficient high-quality evidence, and heterogeneity of participants, interventions (i.e., the applied KT technique) and outcome measures. The efficacy of using KT for ankle sprains may be different depending on the design of the clinical trials. Second, the intervention that is combined with KT may affect the results. In previous studies that showed a positive add-on effect of KT for ankle sprain [28, 34], KT was combined with physiotherapy. However, we selected acupuncture because KT is mainly used with acupuncture in Korean medicine. Third, we applied the ankle meridian tendino-musculature and eight-shape-form of KT treatment, which was used in a previous study [20]. However, several different techniques have been adopted in previous studies that show a positive effect of KT on ankle sprains, whereas only one was used in our study [28, 34–36]. Fourth, the inclusion criteria of this study included the occurrence of grade I or II ALAS within the previous 7 days. The absence of high-quality evidence to inform effective management of ankle sprains in acute care settings is related largely to perceptions that grade I and II ankle sprains are relatively benign injuries [37]. In a previous randomized controlled trial there was no significant add-on effect of supervised physiotherapy on usual care in patients with grade I and II ankle sprains [38].

This study had some limitations. First, we adopted a single outcome assessor-blinding approach because sham treatment was impossible given the characteristics of KT application. This limitation may have led to a bias in the results of the study. Second, this study was performed to investigate the add-on effect of KT for ankle sprains by comparing AcuKT with acupuncture alone. However, there are various conventional treatments (e.g., usual care and physiotherapy) for ankle sprain. Therefore, studies investigating the add-on effect of KT with various conventional treatments or the effect of KT alone may be beneficial. Third, we did not investigate the add-on effect of KT through various ankle KT methods. There are several different techniques for treating lateral ankle sprains. However, we used only ankle meridian tendino-musculature and the eight-shape form of KT treatment. Thus, further studies of an effective treatment method with KT seem necessary. Fourth, our findings were limited to acute grade I or II ALAS. Further studies should analyze the effect of KT in patients with chronic stage or grade III ALAS.

## Conclusions

According to our results, there were no significant differences between the two treatments. AcuKT did not show positive add-on effects of KT with acupuncture in terms of pain reduction, edema, recovery of function, activities of daily living, quality of life or relapse of ALAS. We believe the results from our study could have varied greatly depending on subject characteristics (time after injury and level of severity), KT treatment method, and intervention combination. Therefore, additional studies aimed at investigating possible positive add-on effects of KT in combination with different treatments and different settings should be conducted in the future.

## Supplementary information

**Additional file 1.** Consolidated Standards of Reporting Trials (CONSORT) 2010 checklist of information to include when reporting a randomized trial.

## Data Availability

The datasets used and/or analyzed during the current study are available from the corresponding author on reasonable request.

## Abbreviations

AcuKT: Acupuncture combined with kinesiotape
ALAS: Acute lateral ankle sprain
ANOVA: analysis of variance
CONSORT: Consolidated Standards of Reporting Trials
EQ-5D-5 L: European Quality of Life Five Dimension-Five Level Scale
FAOS: Foot and Ankle Outcome Score
IRB: Institutional Review Board
KT: Kinesiotape
SPIRIT: Standard Protocol Items: Recommendations for Interventional Trials
VAS: Visual analog scale
